# Enhancing Protective Efficacy of Poultry Vaccines through Targeted Delivery of Antigens to Antigen-Presenting Cells

**DOI:** 10.3390/vaccines6040075

**Published:** 2018-11-15

**Authors:** Angita Shrestha, Jean-Remy Sadeyen, Munir Iqbal

**Affiliations:** 1 The Pirbright Institute, Ash Road, Pirbright, Woking GU24 0NF, Surrey, UK; angita.shrestha@pirbright.ac.uk (A.S.); jean-remy.sadeyen@pirbright.ac.uk (J.-R.S.); 2 Department of Zoology, University of Oxford, Oxford OX1 2JD, UK

**Keywords:** antigen-presenting cell, dendritic cell, antigen targeting, antigen targeted vaccine, nanoparticles

## Abstract

Avian viral diseases including avian influenza, Marek’s disease and Newcastle disease are detrimental to economies around the world that depend on the poultry trade. A significant zoonotic threat is also posed by avian influenza viruses. Vaccination is an important and widely used method for controlling these poultry diseases. However, the current vaccines do not provide full protection or sterile immunity. Hence, there is a need to develop improved vaccines. The major aim of developing improved vaccines is to induce strong and specific humoral and cellular immunity in vaccinated animals. One strategy used to enhance the immunogenicity of vaccines is the selective delivery of protective antigens to antigen-presenting cells (APCs) including dendritic cells, macrophages and B cells. APCs have a central role in the initiation and maintenance of immune responses through their ability to capture, process and present antigens to T and B cells. Vaccine technology that selectively targets APCs has been achieved by coupling antigens to monoclonal antibodies or ligands that are targeted by APCs. The aim of this review is to discuss existing strategies of selective delivery of antigens to APCs for effective vaccine development in poultry.

## 1. Introduction

For the past two decades, poultry production and trade have been on the rise globally as poultry meat continues to be one of the most popular animal meats for consumption by man [1]. Currently over 55 billion birds are raised for meat and eggs, with chicken meat accounting for approximately 90% of total poultry meat production [2]. However, poultry continue to be at risk of morbidity and mortality due to infection with bacteria such as *Salmonella* and viruses e.g., avian influenza A virus (IAV), Marek’s disease virus (MDV) and Newcastle disease virus (NDV), which result in significant reductions to the poultry trade and zoonotic infection [3,4,5,6].

Due to the widespread prevalence of these bacteria and viruses, vaccination has become key to controlling them, in addition to surveillance, biosecurity, culling, and restrictions to intra- and international movement [7]. However, the performance of current vaccines remains suboptimal and these pathogens continue to circulate in vaccinated poultry flocks. Thus, there is a need to develop more effective vaccines that induce sterile immunity. One key hurdle in developing such vaccines is eliciting a strong and long-lasting humoral and cellular immune response. To achieve this, we can specifically target antigens to antigen-presenting cells (APCs). Recently, advancements have been made in the fields of molecular biology, cellular biology, immunology and vaccinology, which have led to the development of improved antigen delivery and targeting strategies [8,9]. The targeting strategy determines the intracellular route taken by the antigen into the cell, thus affecting the efficiency of antigen presentation via the major histocompatibility complex (MHC) I and MHC II pathways [10,11,12]. In this review, we will discuss potential strategies of targeting antigens to APCs for effective vaccine development for poultry. We will also briefly discuss novel antigen delivery methods.

## 2. Avian Antigen-Presenting Cells (APCs)

Avian APCs, like mammalian APCs, include B cells, macrophages and dendritic cells (DCs). However, studies have shown that bursectomised birds lacking B cells are able to produce normal T cell responses. Hence, B cells could be less important for antigen presentation in birds [13]. Furthermore, birds lack lymph nodes, the primary site for antigen presentation in mammals [14]. It is thought that heterophils (the chicken functional equivalent of the mammalian neutrophil) and thrombocytes (homologous in function to mammalian platelets, which are absent in chickens) could also possess an ability to present antigens [14]. DCs are the most potent professional APCs in birds and act as a link between innate and acquired immunity [7]. DCs can initiate adaptive immune responses by processing and presenting antigens to T cells. The maturation state of DCs can affect the resultant immune response. Immature migratory DCs behave as antigen-capturing cells to scan the microenvironment for antigens. Upon encountering antigens, they exhibit functional and morphological changes, called maturation, which optimises antigen processing and maximises antigen presentation to naïve T cells [15]. The maturation process involves redistribution of MHC molecules from intracellular endocytic compartments to the cell surface, increase in the expression of co-stimulatory molecules required for the T cell activation, secretion of distinct cytokines and chemokines, cytoskeletal reorganisation and morphological changes including the proliferation of dendrites from the membrane of DCs [16]. Some vaccine adjuvants can induce DC maturation to improve antigen processing and presentation [17].

Comparative gene expression profiling has been used to investigate similarities between immune cell subsets of chickens, humans and mice [18]. Although less work has been reported on chicken DCs, it has been predicted that the antigen processing and presentation pathways are likely to be similar to those of mammals [14]. Matured DCs process exogenous antigens and cytosolic antigens (endogenous) in different ways [19]. Upon encountering exogenous antigens, DCs utilise an endocytic pathway to internalise them into an early endosome. The early endosome matures into a late endosome and fuses with a lysosome that contains proteases and hydrolases. These enzymes degrade antigens into small peptides, which are processed and loaded onto MHC II molecules. MHC II-peptide complexes are recognised by CD4^+^ T cells, which mature and differentiate into T helper cell 1 (Th1) or T helper cell 2 (Th2) cells. Th1 predominantly facilitates a pro-inflammatory response, which involves secretion of interferons (typically IFN-γ) and tumour necrosis factor-α (TNFα), which activate macrophages and natural killer (NK) cells. Th2 cells have an anti-inflammatory role by secreting cytokines e.g., interleukins (IL4, IL10, IL13), which stimulate B cells to produce antibodies [19,20,21]. Furthermore, some pathogens including viruses and bacteria, can be internalised into cells via the non-endocytic pathway. This follows processing of antigens through proteasomal proteolysis, translocation into the endoplasmic reticulum (via Transporter Associated with antigen Processing (TAP)) for further trimming and loading of optimised peptides onto MHC I molecules. These MHC I-peptide complexes are recognised by CD8^+^ T cells, which have cytotoxic activity to kill the infected cells directly [19,20,21].

## 3. Targeting Antigens to Specific APC Receptors

In vaccine development for mammals, antigens targeted to APCs using ligand- or antibody-based methods have shown higher efficacy than their untargeted counterparts [22]. Similar strategies could be applied in developing poultry vaccines and we discuss these below.

### 3.1. Ligand-Based APC Targeting

**Figure 1:**
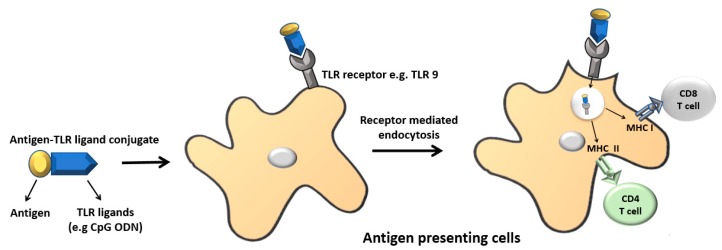
Illustration of ligand-based antigen targeting. Pattern recognition receptor (PRR) ligands can be conjugated to the antigen chemically or recombinantly. In most cases, Toll-like receptor (TLR) ligands have been used for antigen targeting. The TLR ligands have dual functions. They facilitate the targeting of antigens to antigen presenting cells (APCs) and also act as adjuvants. Once the antigen–PRR ligand conjugate binds to the respective receptor on antigen presenting cells, it undergoes receptor mediated endocytosis and is presented either via the MHC I or MHC II pathways. CpG ODN: CpG oligodeoxynucleotide.

In ligand-based APC targeting, antigens are conjugated to the ligand of pattern recognition receptors (PRR). This results in antigen–PRR ligand conjugate vaccines (Figure 1). The conjugated ligand is targeted by the associated PRR on the surface of APCs, which in turn ensures that the APC processes the antigen. There are different PRRs that could be selected when designing antigen–PRR ligand conjugate vaccines depending upon the desired immune response. Out of the four PRR families: Toll-like receptors (TLRs), nucleotide-binding oligomerization domain-like receptors (NLRs), retinoic acid-inducible gene I-like helicases receptors (RLRs), and C-type lectin receptors (CLRs), the TLRs have been extensively used in producing antigen-PRR ligand conjugate vaccines in mammals [9].

TLRs are transmembrane receptors localised on either the cell surface or on endosomal vesicles. They recognise evolutionary conserved molecular motifs (pathogen-associated molecular patterns (PAMPs)) of infectious microbes such as bacterial cell wall components such as lipopolysaccharides (LPS), peptidoglycan, lipopeptides, flagellin, bacterial DNA and viral double-stranded RNA [23]. To date, 13 TLRs have been identified in mammals [24]. In chickens, 10 TLRs have been identified [25,26]. Due to the wide range of functions exhibited by TLRs in avian host defence, they are regarded as, a key target for vaccine development. There have been numerous studies where TLR ligands have been used as adjuvants in vaccine development in poultry [27]. These TLR ligands are promising candidates for ligand-based APC targeting.

Exciting research has recently demonstrated the ability of TLR2 to enhance both direct and cross-presentation of antigens coupled to TLR2 targeting lipid moieties. They have been shown to induce balanced Th responses and to promote the development of antibody responses [28]. It has been demonstrated that TLR2 is involved in the generation and longevity of antibody secreting cells (ASC) [29]. TLR2 is usually described as the TLR recognising the largest range of ligands. These include various bacterial cell wall components such as lipoproteins, peptidoglycan (PGN), LPS, lipoteichoic acids (LTA) [28]. In a study, the effect of three TLR2 ligands: lipomannan, synthetic lipopeptide tri-palmitoyl-S-glyceryl cysteine (Pam_3_C)SK_4_ and fibroblast-stimulating lipopeptide (FSL)-1 on chicken splenocytes were compared. (Pam_3_C)SK_4_ induced high IL-1b responses while (FSL)-1 induced an early and prolonged expression of IL8 [30]. In another study, stimulation of chicken splenocytes by (Pam_3_C)SK_4_ not only produced Th1-associated cytokines IFN-γ and IL-12 but also Th2-associated cytokine IL-4 [31]. Furthermore, outer membrane lipoprotein I (Opr I) has been shown to bind in vitro and in vivo to the epithelial cells of the trachea and the small intestine of chickens. This suggests that Opr I lipoprotein can be used as a ligand to deliver antigens to chicken mucosal surfaces [32].

TLR ligands have been used as adjuvants alongside vaccines for various viral diseases in chickens, for example infectious bursal disease virus (IBDV), avian IAV, NDV and MDV [27]. It has been shown that chickens immunised intramuscularly with formalin-inactivated avian IAV H5N2 along with *Salmonella enterica* flagellin (TLR5 ligand) developed enhanced titres of influenza-specific Immunoglobulin A (IgA) compared to those given formalin-inactivated H5N2 alone. This vaccine formulation also enhanced the proliferation of chicken splenocytes [33]. In addition, when inactivated H5N1 vaccine was administered in chickens with either CpG oligonucleotides (ODN) or poly I:C (ligands for TLR21 and TLR3, respectively), significant levels of secretory IgA and anti- avian IAV-specific IgG were induced in the respiratory tract and serum respectively [34]. Similarly, when CpG-ODN (TLR21 ligand) was used with inactivated NDV vaccine (La Sota strain), it enhanced the levels of serum IgG and secretory IgA, along with induction of T cell proliferation [35]. Moreover, CpG ODN administered with DNA vaccines expressing VP2 proteins of IBDV has been shown to increase the antibody titres and survival rate in chickens following lethal challenge with IBDV [36]. Hence, CpG ODN could be a potential TLR ligand to target antigens to chicken APCs. In rabbit and mouse models, various antigen-TLR-ligand conjugated vaccines have been developed to target TLR9, which is a functional homologue of chicken TLR21 [9].

### 3.2. Antibody-Based APC Targeting

**Figure 2:**
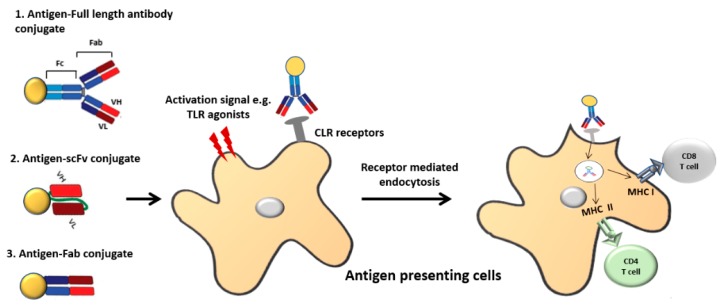
Illustration of antibody-based antigen targeting. The full antibody or antibody fragments such as Fab (fragment antigen binding), scFv (single chain fragment variable antibody) can be conjugated to the antigen chemically or recombinantly. In most cases, C-type lectin receptors (CLR) have been targeted for this purpose. Dendritic cell (DC) activation signals like Toll-like receptor (TLR) agonists are often required for protective immunity. Once the antigen–antibody complex binds to the respective receptor on antigen presenting cells, it undergoes receptor mediated endocytosis and is presented either via the MHC I or MHC II pathway.

Antibodies can target specific antigens that are expressed only on the surface of diseased cells, or highly expressed on the diseased cells compared to the healthy cells. In cancer therapy, this concept has been utilised when treating tumours with antibodies targeting cancer cell receptors conjugated to toxins or radionucleotides to selectively deliver cytotoxic molecules [12]. Antibody targeted vaccines (ATV) similarly exploit the targeting properties of antibodies whereby antibodies are used to deliver a cargo of antigens to APCs. Targeting antibody conjugated antigens to the specific endocytic receptors on APCs could prevent unspecific uptake of antigens by irrelevant non-immune cells, hence it increases amount of antigens reaching the APCs [12]. Antibody-based targeting is carried out either by conjugating antigens to mAbs specific for selected APC surface molecules or by genetic engineering in which the antigen is fused to different antibody fragments such as single chain fragment variable (scFv) or fragment antigen binding (Fab), specific for the APC receptors (Figure 2). The antigen used can either be the entire protein or small disease-specific peptides. The coupling of antigen to antibody can be carried out either by chemical linkage or by production of recombinant antibody-antigen fusion constructs [12]. There are several advantages of using recombinant antigen coupled antibodies over chemically-conjugated antibodies. Firstly, it is possible to genetically modify the targeting antibodies. This allows us to modify their solubility (by changing amino acid composition), their ability to fix complement components (by changing glycosylation sites) and their binding to Fc receptors (by changing glycosylation or using Fab or scFv). Moreover, multiple antigens or epitopes from different antigens can be assembled into one construct allowing the induction of a broader immune response [11].

The initial ATV studies focused on inducing a humoral immune response whereby model antigens such as egg avidin and egg lysozyme were targeted to MHC molecules and Fc receptors in mice (without adjuvants) [37,38]. The antigen-specific antibody titres generated were comparable to immunisations with antigens emulsified in complete Freund’s adjuvant. These initial studies demonstrated that robust antibody responses to ATVs could be obtained with a very low concentration of antigen [37,38,39]. Currently, ATVs are being exploited for their ability to potentiate an immune response against various targets, including viruses.

In mammals, research into antibody-based targeting has primarily focused on receptors such as CLRs, integrins and Fc receptors. Among CLRs, dendritic cell receptor for Endocytosis-205 (DEC-205), NK lectin group receptor-1 (DNGR-1) and dendritic cell-specific intercellular adhesion molecule-3-grabbing non-integrin (DC-SIGN) are the major receptors being targeted [11]. DEC-205 is a CLR that belongs to the family of mannose receptor (MR). It is internalised by means of coated pits and vesicles and traffics to endosomal compartments which are rich in MHC II molecules. This receptor is shown to enhance antigen presentation via the MHC II pathway and is also involved in the cross-presentation [40]. Chicken DEC-205 has a highly conserved structure with 51% and 48% amino acid sequence similarity to human and mouse DEC-205, respectively [40]. In mammals, DEC-205 is primarily expressed on DCs and thymic cortical epithelium. The chicken DEC-205 is one of the important DC markers along with cluster of differentiation 45 (CD45), CD83, CD11c and colony stimulating factor 1 receptor (CSF1R) [41]. The first antibody targeting study in chicken was directed towards DEC-205 receptor. Avian IAV H5N2 haemagglutinin (HA) was chemically conjugated to anti-chicken DEC-205 antibody. Indirect enzyme-linked immunosorbent assay (ELISA) was used to measure HA specific antibody titre in chicken serum samples by coating the plates with recombinant HA. A single dose of this ATV was shown to be sufficient to elicit a strong antibody response in chickens as early as fourteen days after priming [42]. Several mammalian studies have also shown that targeting antigens to DEC-205 induced a strong humoral response, both in presence and absence of adjuvants [43,44]. The CDX-1401 vaccine (Celldex Therapeutics Inc., New Haven, Connecticut, USA) was used in the first phase I study of a protein vaccine targeting DCs in vivo in human. This was an anti-cancer vaccine and comprised of tumour antigen NY-ESO-1 in full-length fused to anti-DEC-205 antibody. This vaccine was proven to be safe in humans and induced both humoral and cellular response against the tumour antigens [45].

In mice, CD11c receptor which belongs to the family of β_2_ integrins is considered as an effective immune receptor and is expressed in all DC subsets at high levels [46]. In mice, the in vivo targeting of antigens to DCs via CD11c has been shown to result in the efficient processing and presentation of antigens on both MHC I and MHC II molecules, inducing robust CD4^+^ and CD8^+^ T cell responses [47]. In addition, antigen-containing liposomes coated with CD11c antibody have been shown to induce Cytotoxic T lymphocyte (CTL) responses and protect mice against subsequent tumour challenge, provided LPS or IFN-γ was co-administered [48]. Several groups have applied CD11c antibodies to selectively stain chicken DCs, NK cells and macrophages. It has been reported that bursal secretory dendritic cells (BSDC) express putative CD11c [41]. Thus, CD11c is thought to be a potential receptor for vaccine target in chickens. Similarly, chB6 (Bu-1) molecule has been identified as a unique B cell marker in chickens and is not found in mammals [49]. It is a highly glycosylated type I transmembrane protein and thought to be important for B cell development [50]. Hence, antibodies targeting chB6 could potentially be important for activation of B cells in chickens.

#### Different Parameters of Targeting Antibody That Affect Immunogenicity

There are several factors that influence the outcome of the immune response induced by APCs, when using an antibody to target an antigen. These factors are either related to the different types of APCs being targeted, the choice of the target receptor or the specific antibody used. Regarding the influence by the specific antibody used, it has been shown that the intracellular route for internalised antigens is dependent on whether the mAb recognises the stalk region or the lectin domain of DC-SIGN [51]. In mice, some anti-Clec9A mAbs were reported to induce a strong humoral responses in the absence of adjuvants, whereas other anti-Clec9A mAbs were unable to induce humoral response in the absence of adjuvants [52,53]. This could relate to issues with affinity and epitope specificity. Another feature of the antibody that might affect the immune response is its isotype. Antigens targeted to Clec9A using rat IgG2A mAb resulted in strong antibody responses in absence of adjuvants. On the other hand, when rat IgG1 mAb was used to target Clec9A, DC activation factor was required to induce a strong antibody response. This was suggested to be due to the lack of T cell epitopes in rat IgG1 [54]. Also, the isotype of the targeted antibody could influence the persistence of the antibody–antigen complex in the bloodstream, affecting the chances of the constructs reaching the specific APCs [55]. Furthermore, antibodies typically display variable capacity for initiating a signaling response within APCs. These signaling cascades play an important role in the efficiency of the response to an antigen. Large-scale gene expression profiling was used for characterising the signaling mediated by DC-SIGN upon activation by DC-SIGN-specific antibody; some antibodies were able to induce cell signaling whereas others were not [56]. There could be factors like the affinity of the antibody for the APC target molecule or the specific epitope bound that can affect the immunogenicity.

### 3.3. Nanoparticle-Based APC Targeting

**Figure 3:**
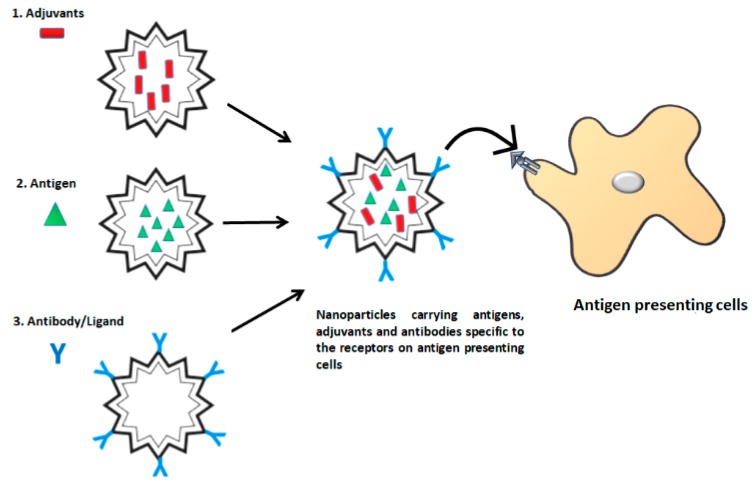
Illustration of nanoparticle-based antigen targeting. Various nanoparticles such as poly-d,l-lactide-co-glycolide (PLGA), polylactic acid (PLA), chitosan, liposomes and virus-like particles (VLPs) have been used for antigen targeting. These nanoparticles can be decorated on their surface with antibodies or ligands that can bind specifically to the antigen presenting cell receptors.

Methods involving nanoparticles offer a novel approach for in situ targeting of antigens to APCs. They involve encapsulating both antigens and adjuvants within delivery vehicles (Figure 3) [57]. By co-delivering both antigens and adjuvants in the same compartment, these nano-carriers ensure that only APCs exposed to antigens receive the activation signal and prevent any non-specific activation of other APCs [21]. Nanoparticle delivery systems include a wide range of nano-scale size materials (<1 µm) such as polymeric particles, liposomes, virus-like particles (VLPs), nanocrystals, immune-stimulating complexes (ISCOMs) and virosomes. In order to target nanoparticles encapsulating specific antigen to the APC receptor, the surface of nanoparticles is decorated with antibodies or carbohydrate ligands that bind specifically to the APC receptor [21]. Polymer nanoparticles and liposomes can be coated with antibodies by PEGylation or avidin-biotin interactions while VLPs are generally engineered to express receptor ligands [9]. It has been shown that nanoparticles coated with DC receptor-specific antibodies were more efficient in targeting human DCs in comparison to the nanoparticles coated with carbohydrate ligands [58]. These nanoparticles are efficiently taken up by DCs because their size and particulate structure are similar to that of pathogens [59]. They can also induce long-lasting immune responses by delivering antigens in a slow and sustained manner. Furthermore, the release properties of the nanoparticles can be easily controlled by regulating their physicochemical characteristics (size, surface charge, biomaterial composition, hydrophobicity/hydrophilicity). In addition, antigens encapsulated in nanoparticles can be protected from enzymatic degradation, improving their half-life and solubility [60]. Antigens delivered by nanoparticles are internalised via various endocytic pathways. For example, particles ~1 µm are internalised via micropinocytosis, ~120 nm are internalised by receptor-mediated clathrin endocytosis, and ~90 nm are internalised by caveolae-mediated lipid rafts or caveolin-independent endocytosis [61]. These different internalisation routes can determine the intracellular fate of antigen processing and subsequent T cell activation.

Out of several nanoparticles, polymeric nanoparticles such as poly-d,l-lactide-co-glycolide (PLGA), polylactic acid (PLA), chitosan, liposomes and VLPs are extensively studied for drug and antigen delivery system. Two chitosan derivatives, *O*-2′-hydroxypropyltrimethyl ammonium chloride chitosan and *N*-2-hydroxypropyl trimethyl ammonium chloride chitosan, have been used to make nanoparticles as a mucosal delivery vehicle for live attenuated ND vaccines. The release of NDV was effective in both systems with stronger cellular, humoral and mucosal immune response [62]. Liposomes on the other hand, are spherical, uni- or multi-lamellar, nano- or micro-sized vesicles composed of phospholipid bilayers. They are biodegradable and non-toxic [63]. They can incorporate viral envelope glycoproteins (haemagglutinin and neuraminidase of influenza virus) to form virosomes [64]. Hydrophilic antigens are incorporated within the aqueous hollow cavity, whereas hydrophobic antigens are inserted into the bilayer [63]. Liposomes can be targeted to APCs by introducing receptor ligands into the bilayer by metal chelating linkage of His-tagged antibodies to the bilayer, or by first introducing protein A into the bilayer and then attaching receptor-specific antibodies to protein A [65]. Liposomes have been used as adjuvanted vaccine candidates due to their ability to induce humoral and CTL responses against the encapsulated antigens [65]. In various studies ND vaccines have been developed using phosphatidylcholine/cholesterol liposomes in order to deliver antigens. Such liposomal vaccines have shown enhanced immune response with higher antibody titre and T-cell, B-cell proliferation in chickens [65].

VLPs (pseudovirions) are self-assembling nanoparticles composed of one or more viral structural proteins; capsid and/or envelope proteins and lacking an infectious viral genome. Thus, they are potentially safer alternatives to live, attenuated and inactivated vaccines. They can be derived from different viruses with sizes ranging from 20 nm to 800 nm and there are currently well-developed manufacturing processes to ensure consistency [66]. Antigens can be incorporated into the insertion sites of VLPs by genetic fusion (chimeric VLP) or by in vitro chemical conjugation (conjugated VLPs) [67]. The naturally optimised size, the highly ordered and repetitive structure, the charge surface coupled with immunogenic properties and adjuvanticity make the VLP an attractive antigen delivery system, vaccine candidate and targeted drug carrier. VLPs are efficiently taken up by APCs for both MHC I and MHC II presentation. It has been shown that the highly repetitive surface structures of VLPs are able to induce DC and B cell maturation by triggering TLRs and cross-linking B cell receptors, respectively [68]. Thus, a strong B and T cell immune response can be stimulated. Various studies have been carried out to develop effective VLP vaccines to treat avian viral diseases. Chimeric VLP vaccines have been shown to confer protection in chickens against high pathogenic avian influenza (HPAI) subtype H5N1 and velogenic NDV. Such chimeric VLPs also allowed a strategy of differentiating infected from vaccinated animals (DIVA) [69]. Similarly, another study showed that H5/H7/H9/N1/gag VLPs could induce immune responses against heterologous H5, H7 and H9 virus challenges, providing a platform for the development of broadly protective avian IAV vaccines [70]. Furthermore, there have also been reports of potential VLP vaccines against IBDV [71].

## 4. Other Antigen Delivery Systems

### 4.1. Viral Vectors

**Figure 4:**
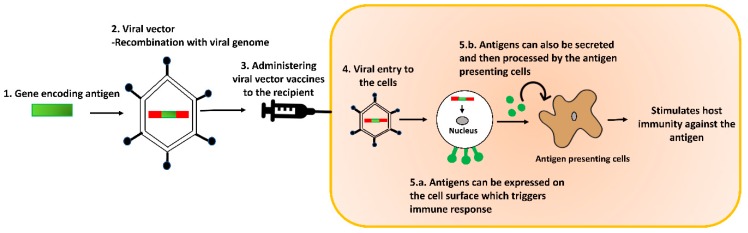
Illustration of viral vector vaccine delivery method. The gene encoding the antigen of interest can be introduced into the viral genome via recombination. The recombinant viral vectors can be administered to the recipient. Upon entry into the recipient cells, they release the viral genome into the cytoplasm. This results in the in vivo expression of the encoded antigen. The antigen can either be expressed on the cell surface, or secreted and then processed by antigen-presenting cells.

Viral-based delivery systems consist of either live attenuated viruses or genetically modified replication defective viruses that drive the expression of recombinant antigens integrated into their genome following replication in the cells they invade (Figure 4). This facilitates antigen presentation by the MHC I pathway [63]. Recombinant viral vectors can be a potential platform for delivering antigens targeted with APC specific antibodies, as they allow long-term antigen production in vivo. Viral vectors such as adenovirus, vaccinia virus, alphavirus, herpes simplex virus (HSV), have been exploited as recombinant viral vaccines for livestock [72]. Viral vectors have potential to act as adjuvants [63]. They also allow easy differentiation of infected and vaccinated animals. Thus, it is easier to monitor infection even in vaccinated animals [72]. Furthermore, viruses such as herpesvirus of turkey (HVT), NDV, fowl pox virus (FPV), adenovirus, infectious laryngotracheitis virus (ILTV) and MDV have been genetically modified to be used as vaccine vectors for combating multiple avian viral diseases [72]. Despite several advantages, there are certain safety issues to consider when using viral vectors for antigen delivery. Some of these concerns include the possibility of the integration of viral DNA into host chromosomes, reversion to virulence, the transcriptional activation of oncogenes, and hindrance of replication in animals carrying pre-existing immunity against the viral vectors.

### 4.2. Cell-Penetrating Peptides (CPP)

**Figure 5:**
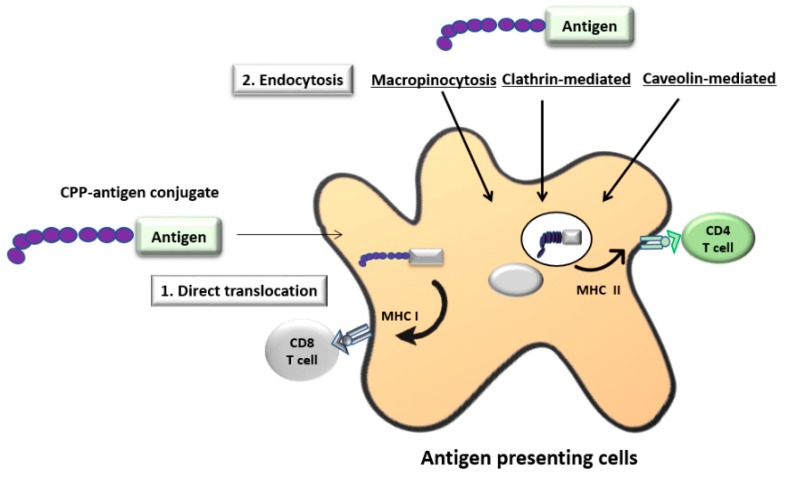
Illustration of the cell-penetrating peptide (CPP) delivery system. CPP are cationic peptides. CPP–antigen conjugates can be designed either chemically or recombinantly. CPP can undergo electrostatic interaction with the negatively charged cell surface glycoproteins under physiological condition. This facilitates the direct translocation of antigens across the cell membrane. Furthermore, CPP–antigen conjugates can also enter the cell by endocytosis. Direct translocation of the CPP–antigen conjugate allows antigen presentation via the MHC I pathway whereas entry by endocytosis facilitates the antigen presentation via the MHC II pathway.

Cell-penetrating peptides (CPP) are a group of cationic peptides (about 4–30 amino acids length) that have the ability to enter the cytoplasm of the cells [73,74]. They are rich in lysine and/or arginine. They can deliver a number of antigens including RNA, DNA, peptides, proteins and drugs into the cell. Transactivator of transcription proteins (TAT) from human immunodeficiency virus (HIV) and penetratin from the *Drosophila antennapedia* domain are the most well-known CPP that have been used to deliver antigens with induction of strong cellular and antibody responses [73]. Illustration of the CPP antigen delivery system is shown in Figure 5. The mechanism of uptake and internalisation of CPP is unknown. However, it is thought that small CPPs can be internalised by both endocytosis and direct translocation across the membrane whereas the large CPP-cargo molecules are internalised only via the endocytic pathway [75,76]. After endocytosis, the CPP-cargo molecules are enclosed in endosomes. Some unknown properties of CPPs allow them to escape from the endosome and diffuse into the cytoplasm [74]. This can enable antigen presentation by MHC I molecules, leading to more efficient CTL responses. Some CPP-antigen complexes can remain in the endosome, fuse with lysosomes, and undergo lysosomal degradation facilitating the MHC II pathway, and enhancing CD4^+^ T-cell response [77]. Furthermore, CPP-antigen complex can be internalised into DCs without the need of receptor targeting molecules (antibodies or ligands). As CPPs are positively charged under physiological conditions, they can undergo electrostatic interaction with the negatively-charged cell surface glycoproteins (GAG: glycosaminoglycans) [78]. This induces internalisation of target antigens to the cytoplasm. The CPP–antigen complex can be designed either by chemical linkage via covalent bonds (amide, thioether or thiol maleimide) or by producing recombinant fusion constructs [76]. Moreover, the basic characteristics of CPPs are similar to nuclear localization sequences (NLS), and thus some CPPs can deliver plasmid DNA and transcription factors into the nucleus [79]. Several studies have suggested that CPPs can be used for DC vaccination against cancer and other infectious diseases by inducing antigen specific CD4^+^ and CD8^+^ T cell responses [80,81]. However, it has been shown that there is no advantage of using CPPs over receptor-specific antibodies in targeting antigens to human DCs [82].

### 4.3. E2 Scaffold System

**Figure 6:**
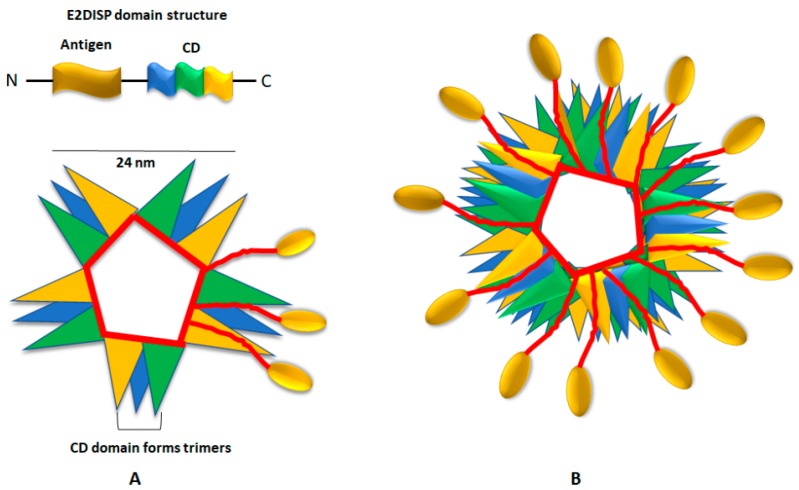
Illustration of the structure of E2 acetyltransferase display system (E2DISP) domain. (**A**) Representation of five catalytic acetyltransferase core domain (CD) trimers (monomers of each trimer is represented by blue, green and yellow colours). The C-terminal CD of E2 can self-assemble into trimers which aggregate to generate a pentagonal dodecahedral protein scaffold (see B). (**B**) Twenty trimers of the E2 polypeptide chain form a pentagonal dodecahedron (60-mer) with icosahedral symmetry. About 60 large polypeptides can be presented on the E2 scaffold as N-terminal fusions to the acetyltransferase CD. These particles can self-assemble in vitro and elicit T-cell and B-cell responses.

This is a novel antigen delivery system based on the pyruvate dehydrogenase (PDH) complex of *Geobacilus stearothermophilus* [63]. The PDH complex is made up of multiple copies of three different enzymes; E1, E2 and E3. E2 is a dihydrolipoyl acyltransferase enzyme composed of three independently folded domains separated by flexible linkers: a lipoyl domain (LD), a peripheral subunit binding domain (PSBD) and a catalytic acetyltransferase core domain (CD). The C-terminal CD of E2 can self- assemble into trimers, which aggregate to generate a pentagonal dodecahedral protein scaffold with icosahedral symmetry resembling a VLP (Figure 6) [83]. The exogenous peptides are displayed on the E2 scaffold by engineering plasmids that can allow the insertion of the exogenous oligonucleotides at the 5’ end of the gene encoding E2 CD. Such engineered E2 core is referred to as the E2 acetyltransferase display system (E2DISP) and it has an ability to display 60 copies of heterologous polypeptides on the E2 core surface [84]. One of the key advantages of this system is that the E2 core domain can naturally display 187 amino acid residues in the form of two folded protein domains (LD and PSBD) and two flexible linkers [63]. This facilitates the display of the full-length protein as antigen, which is more advantageous than displaying smaller peptides in terms of higher epitope diversity for antibody production and T cell induction. Furthermore, the display of 60 copies of an antigen of interest per E2DISP particle can stimulate a stronger immune response by triggering and cross-linking specific B-cell antigen receptor [63]. Studies have demonstrated that epitopes displayed on the E2 surface can elicit both B and T cell responses, indicating that E2DISP particle can process the displayed epitopes via both MHC I and MHC II pathways [85,86,87]. Factors such as stability, non-toxicity, low cost and lack of integration make E2DISP an attractive antigen delivery system.

## 5. Conclusions

Poultry vaccine technology can benefit greatly from approaches that involve targeting antigens to key APCs. Various mammalian studies have shown that targeting antigens to APCs increases the efficacy of vaccines by preventing the unwanted uptake of antigens by irrelevant non-immune cells. This selective delivery increases the amount of antigens reaching target APCs. Hence, lesser vaccine doses can be administered, which is an effective way of inducing rapid and strong immune responses cost-effectively. Vaccine dose sparing is one key advantage for developing poultry vaccines considering the low market value of the individual animals. Efficient in vivo targeting of antigens to APCs is quite challenging. Knowing which receptor to target is important in order to induce the desired immune responses. Considering that there are similarities between avian and mammalian immune systems, we can refer to some of the mammalian studies in order to predict which avian APC receptors could be targeted. However, further research is required for identifying the most responsive avian APCs for antigen targeting. There is also a need to continue exploring novel antigen targeting systems that use various ligands, antibodies and nanoparticles, which transport antigen to avian APCs efficiently and selectively. Addressing the range of current limitations in antigen target/delivery systems will be a step change in the vaccinology field and will help in preventing and controlling infectious diseases affecting both animals and humans.

## References

[1] Food and Agriculture Organisation Meat Market Review. http://www.fao.org/3/I9286EN/i9286en.pdf.

[2] Food and Agriculture Organization of the United Nations Poultry Species, Gateway to Poultry Production and Products. http://www.fao.org/poultry-production-products/production/poultry-species/en/.

[3] Economic and Social Impact of Avian Influenza. http://www.fao.org/docs/eims/upload/211939/economic-and-social-impacts-of-avian-influenza-geneva.pdf.

[4] Antipas B., Kebkiba B., Mopate L.Y. (2012). Epidemiology of Newcastle disease and its economic impact in chad. Eur. J. Exp. Biol..

[5] English B.C., Menard J., Jensen K. (2005). Projected Economic Impacts of an Exotic Newcastle Disease (END) Outbreak in Tennesse.

[6] Fakhrul Islam A., Walkden-Brown S., Groves P., Underwood G. (2008). Kinetics of marek’s disease virus (MDV) infection in broiler chickens 1: Effect of varying vaccination to challenge interval on vaccinal protection and load of MDV and herpesvirus of turkey in the spleen and feather dander over time. Avian Pathol..

[7] Marangon S., Busani L. (2006). The use of vaccination in poultry production. Rev. Sci. Technol..

[8] Apostolopoulos V. (2016). Vaccine Delivery Methods into The Future. Vaccines.

[9] Chen P., Liu X., Sun Y., Zhou P., Wang Y., Zhang Y. (2015). Dendritic Cell Targeted Vaccines: Recent Progresses And Challenges. Hum. Vaccines Immunother..

[10] Apostolopoulos V., Thalhammer T., Tzakos A., Stojanovska L. (2013). Targeting Antigens To Dendritic Cell Receptors For Vaccine Development. J. Drug Deliv..

[11] Pugholm L.H., Varming K., Agger R. (2016). Antibody-Mediated Delivery of antigen to dendritic cells. Immunother. Open Access.

[12] Keler T., He L., Ramakrishna V., Champion B. (2007). Antibody-Targeted Vaccines. Oncogene.

[13] Beal R., Powers C., Davison T., Barrow P., Smith A. (2006). Clearance of enteric salmonella enterica serovar typhimurium in chickens is independent of b-cell function. Infect. Immunity.

[14] Wu Z., Kaiser P. (2011). Antigen presenting cells in a non-mammalian model system, the chicken. Immunobiology.

[15] Singla B., Ghoshal P., Lin H., Wei Q., Dong Z., Csányi G. (2018). Pkcδ-Mediated Nox2 Activation Promotes Fluid-Phase Pinocytosis of Antigens by Immature Dendritic Cells. Front. Immunol..

[16] Verdijk P., van Veelen P., de Ru A., Hensbergen P., Mizuno K., Koerten H., Koning F., Tensen C., Mommaas A. (2004). Morphological changes during dendritic cell maturation correlate with cofilin activation and translocation to the cell membrane. Eur. J. Immunol..

[17] García A., De Sanctis J. (2013). An Overview of Adjuvant Formulations and Delivery Systems. Apmis.

[18] Vu Manh T., Marty H., Sibille P., Le Vern Y., Kaspers B., Dalod M., Schwartz-Cornil I., Quere P. (2014). Existence of Conventional Dendritic Cells in Gallus Gallus Revealed by Comparative Gene Expression Profiling. J. Immunol..

[19] Compeer E., Flinsenberg T., van der Grein S., Boes M. (2012). Antigen Processing And Remodeling of the endosomal pathway: Requirements for antigen cross-presentation. Front. Immunol..

[20] Sadegh-Nasseri S., Kim A. (2015). MHC Class II Auto-Antigen Presentation Is Unconventional. Front. Immunol..

[21] Adiko A., Babdor J., Gutiérrez-Martínez E., Guermonprez P., Saveanu L. (2015). Intracellular Transport Routes for MHC I and Their Relevance for Antigen Cross-Presentation. Front. Immunol..

[22] Sehgal K., Dhodapkar K., Dhodapkar M. (2014). Targeting Human Dendritic Cells in Situ Improve Vaccines. Immunol. Lett..

[23] Janeway C., Medzhitov R. (2002). Innate Immune Recognition. Annu Rev Immunol..

[24] Shi Z., Cai Z., Sanchez A., Zhang T., Wen S., Wang J., Yang J., Fu S., Zhang D. (2010). A Novel Toll-Like Receptor That Recognizes Vesicular Stomatitis Virus. J. Biol. Chem..

[25] Iqbal M., Philbin V., Smith A. (2005). Expression Patterns Of Chicken Toll-Like Receptor mRNA In Tissues, Immune Cell Subsets And Cell Lines. Vet. Immunol. Immunopathol..

[26] Higgs R., Cormican P., Cahalane S., Allan B., Lloyd A., Meade K., James T., Lynn D., Babiuk L., O’Farrelly C. (2006). Induction of a Novel Chicken Toll-Like Receptor Following Salmonella Enterica Serovar Typhimurium Infection. Infect. Immunity.

[27] Gupta S., Deb R., Dey S., Chellappa M. (2014). Toll-Like receptor-based adjuvants: Enhancing the immune response to vaccines against infectious diseases of chicken. Expert Rev. Vaccines.

[28] Basto A., Leitão A. (2014). Targeting TLR2 For Vaccine Development. J. Immunol. Res.

[29] Boeglin E., Smulski C., Brun S., Milosevic S., Schneider P., Fournel S. (2011). Toll-Like Receptor Agonists Synergize With CD40L to induce either proliferation or plasma cell differentiation of mouse B Cells. PLoS ONE.

[30] St. Paul M., Paolucci S., Sharif S. (2013). Characterization of Responses Initiated By Different Toll-Like Receptor 2 Ligands In Chicken Spleen Cells. Res. Vet. Sci..

[31] St. Paul M., Paolucci S., Sharif S. (2012). Treatment with Ligands for Toll-Like Receptors 2 and 5 Induces a Mixed T-helper 1- and 2-Like Response in Chicken Splenocytes. J. Interferon Cytokine Res..

[32] Loots K., Revets H., Goddeeris B. (2008). Attachment of the outer membrane lipoprotein (OprI) of Pseudomonas aeruginosa to the mucosal surfaces of the respiratory and digestive tract of chickens. Vaccine.

[33] Chaung H., Cheng L., Hung L., Tsai P., Skountzou I., Wang B., Compans R., Lien Y. (2012). Salmonella flagellin enhances mucosal immunity of avian influenza vaccine in chickens. Vet. Microbiol..

[34] Liang J., Fu J., Kang H., Lin J., Yu Q., Yang Q. (2013). Comparison of 3 kinds of Toll-like receptor ligands for inactivated avian H5N1 influenza virus intranasal immunization in chicken. Pollut. Sci..

[35] Zhang L., Zhang M., Li J., Cao T., Tian X., Zhou F. (2008). Enhancement of mucosal immune responses by intranasal co-delivery of Newcastle disease vaccine plus CpG oligonucleotide in SPF chickens in vivo. Res. Vet. Sci..

[36] Mahmood M., Siddique M., Hussain I., Khan A., Mansoor M. (2006). Protection capability of recombinant plasmid DNA vaccine containing VP2 gene of very virulent infectious bursal disease virus in chickens adjuvanted with CpG oligodeoxynucleotide. Vaccine.

[37] Carayanniotis G., Barber B. (1990). Characterization of the adjuvant-free serological response to protein antigens coupled to antibodies specific for class II MHC determinants. Vaccine.

[38] Snider D.P., Kaubisch A., Segal D.M. (1990). Enhanced antigen immunogenicity induced by bispecific antibodies. J. Exp. Med..

[39] Snider D.P., Segal D.M. (1987). Targeted antigen presentation using crosslinked antibody heteroaggregates. J. Immunol..

[40] Staines K., Young J., Butter C. (2013). Expression of Chicken DEC205 Reflects the Unique Structure and Function of the Avian Immune System. PLoS ONE.

[41] Nagy N., Bódi I., Oláh I. (2016). Avian dendritic cells: Phenotype and ontogeny in lymphoid organs. Dev. Comp. Immunol..

[42] Jáuregui-Zúñiga D., Pedraza-Escalona M., Espino-Solís G., Quintero-Hernández V., Olvera-Rodríguez A., Díaz-Salinas M., López S., Possani L. (2017). Targeting antigens to Dec-205 on dendritic cells induces a higher immune response in chickens: Hemagglutinin of avian influenza virus example. Res. Vet. Sci..

[43] Bonifaz L., Bonnyay D., Mahnke K., Rivera M., Nussenzweig M.C., Steinman R.M. (2002). Efficient Targeting of Protein Antigen to the Dendritic Cell Receptor DEC-205 in the Steady State Leads to Antigen Presentation on Major Histocompatibility Complex Class I Products and Peripheral CD8^+^ T Cell Tolerance. J. Exp. Med..

[44] Pugholm L., Petersen L., Søndergaard E., Varming K., Agger R. (2015). Enhanced Humoral Responses Induced by Targeting of Antigen to Murine Dendritic Cells. Scand. J. Immunol..

[45] Dhodapkar M., Sznol M., Zhao B., Wang D., Carvajal R., Keohan M., Chuang E., Sanborn R., Lutzky J., Powderly J. (2014). Induction of Antigen-Specific Immunity with a Vaccine Targeting NY-ESO-1 to the Dendritic Cell Receptor DEC-205. Sci. Transl. Med..

[46] Beyer M., Wang H., Peters N., Doths S., Koerner-Rettberg C., Openshaw P., Schwarze J. (2005). The beta2 integrin CD11c distinguishes a subset of cytotoxic pulmonary T cells with potent antiviral effects in vitro and in vivo. Respir. Res..

[47] Castro F., Tutt A., White A., Teeling J., James S., French R., Glennie M. (2008). CD11c provides an effective immunotarget for the generation of both CD4 and CD8 T cell responses. Eur. J. Immunol..

[48] Van Broekhoven C., Parish C., Demangel C., Britton W., Altin J. (2004). Targeting Dendritic Cells with Antigen-Containing Liposomes. Cancer Res..

[49] Tregaskes C.A., Bumstead N., Davison T.F., Young J.R. (1996). Chicken B-cell marker chB6 (Bu-1) is a highly glycosylated protein of unique structure. Immunogenetics.

[50] Funk P.E., Tregaskes C.A., Young J.R., Thompson C.B. (1997). The avian chB6 (Bu-1) alloantigen can mediate rapid cell death. J. Immunol..

[51] Tacken P., Ginter W., Berod L., Cruz L., Joosten B., Sparwasser T., Figdor C., Cambi A. (2011). Targeting DC-SIGN via its neck region leads to prolonged antigen residence in early endosomes, delayed lysosomal degradation, and cross-presentation. Blood.

[52] Li J., Ahmet F., Sullivan L., Brooks A., Kent S., De Rose R., Salazar A., Reis e Sousa C., Shortman K., Lahoud M. (2015). Antibodies targeting Clec9A promote strong humoral immunity without adjuvant in mice and non-human primates. Eur. J. Immunol..

[53] Lahoud M., Ahmet F., Kitsoulis S., Wan S., Vremec D., Lee C., Phipson B., Shi W., Smyth G., Lew A. (2011). Targeting Antigen to Mouse Dendritic Cells via Clec9A Induces Potent CD4 T Cell Responses Biased toward a Follicular Helper Phenotype. J. Immunol..

[54] Joffre O., Sancho D., Zelenay S., Keller A., Reis e Sousa C. (2010). Efficient and versatile manipulation of the peripheral CD4^+^ T-cell compartment by antigen targeting to DNGR-1/CLEC9A. Eur. J. Immunol..

[55] Caminschi I., Shortman K. (2012). Boosting antibody responses by targeting antigens to dendritic cells. Trends Immunol..

[56] Hodges A., Sharrocks K., Edelmann M., Baban D., Moris A., Schwartz O., Drakesmith H., Davies K., Kessler B., McMichael A. (2007). Activation of the lectin DC-SIGN induces an immature dendritic cell phenotype triggering Rho-GTPase activity required for HIV-1 replication. Nat. Immunol..

[57] Paulis L., Mandal S., Kreutz M., Figdor C. (2013). Dendritic cell-based nanovaccines for cancer immunotherapy. Curr. Opin. Immunol..

[58] Cruz L., Tacken P., Pots J., Torensma R., Buschow S., Figdor C. (2012). Comparison of antibodies and carbohydrates to target vaccines to human dendritic cells via DC-SIGN. Biomaterials.

[59] Park Y., Lee S., Kim Y., Lee M., Cha G., Jung I., Kang T., Han H. (2013). Nanoparticle-Based Vaccine Delivery for Cancer Immunotherapy. Immune Netw..

[60] Gregory A., Titball R., Williamson D. (2013). Vaccine delivery using nanoparticles. Front. Cell. Infect. Microbiol..

[61] Zhao L., Seth A., Wibowo N., Zhao C., Mitter N., Yu C., Middelberg A. (2014). Nanoparticle vaccines. Vaccine.

[62] Dimitrov K., Afonso C., Yu Q., Miller P. (2017). Newcastle disease vaccines—A solved problem or a continuous challenge?. Vet. Microbiol..

[63] Trovato M. (2015). Novel antigen delivery systems. World J. Virol..

[64] Huckriede A., Bungener L., Stegmann T., Daemen T., Medema J., Palache A., Wilschut J. (2005). The virosome concept for influenza vaccines. Vaccine.

[65] Schwendener R. (2014). Liposomes as vaccine delivery systems: A review of the recent advances. Ther. Adv. Vaccines.

[66] Kushnir N., Streatfield S., Yusibov V. (2012). Virus-like particles as a highly efficient vaccine platform: Diversity of targets and production systems and advances in clinical development. Vaccine.

[67] Jain N., Sahni N., Kumru O., Joshi S., Volkin D., Russell Middaugh C. (2015). Formulation and stabilization of recombinant protein based virus-like particle vaccines. Adv. Drug Deliv. Rev..

[68] Noad R., Roy P. (2003). Virus-like particles as immunogens. Trends Microbiol..

[69] Noh J., Park J., Lee D., Yuk S., Kwon J., Lee S., Lee J., Park S., Choi I., Song C. (2016). Chimeric Bivalent Virus-Like Particle Vaccine for H5N1 HPAI and ND Confers Protection against a Lethal Challenge in Chickens and Allows a Strategy of Differentiating Infected from Vaccinated Animals (DIVA). PLoS ONE.

[70] Pushko P., Tretyakova I., Hidajat R., Zsak A., Chrzastek K., Tumpey T., Kapczynski D. (2017). Virus-Like Particles Displaying H5, H7, H9 Hemagglutinins and N1 Neuraminidase Elicit Protective Immunity To Heterologous Avian Influenza Viruses In Chickens. Virology.

[71] Choi K. (2016). A Virus-like Particle Vaccine against Infectious Bursal Disease Virus: Potential Uses and Applications. Br. J. Virol..

[72] Baron M., Iqbal M., Nair V. (2018). Recent advances in viral vectors in veterinary vaccinology. Curr. Opin. Virol..

[73] Lim S., Koo J., Choi J. (2016). Use of Cell-Penetrating Peptides in Dendritic Cell-Based Vaccination. Immune Netw..

[74] Erazo-Oliveras A., Muthukrishnan N., Baker R., Wang T., Pellois J. (2012). Improving the Endosomal Escape of Cell-Penetrating Peptides and Their Cargos: Strategies and Challenges. Pharmaceuticals.

[75] Ramsey J., Flynn N. (2015). Cell-penetrating peptides transport therapeutics into cells. Pharm. Ther..

[76] Koren E., Torchilin V. (2012). Cell-penetrating peptides: Breaking through to the other side. Trends Mol. Med..

[77] El-Sayed A., Futaki S., Harashima H. (2009). Delivery of Macromolecules Using Arginine-Rich Cell-Penetrating Peptides: Ways to Overcome Endosomal Entrapment. AAPS J..

[78] Ziegler A. (2008). Thermodynamic studies and binding mechanisms of cell-penetrating peptides with lipids and glycosaminoglycans. Adv. Drug Deliv. Rev..

[79] Zaro J., Vekich J., Tran T., Shen W. (2009). Nuclear Localization of Cell-Penetrating Peptides Is Dependent On Endocytosis Rather Than Cytosolic Delivery in CHO Cells. Mol. Pharm..

[80] Wadia J., Dowdy S. (2005). Transmembrane delivery of protein and peptide drugs by TAT-mediated transduction in the treatment of cancer. Adv. Drug Deliv. Rev..

[81] Foerg C., Merkle H. (2008). On The Biomedical Promise of Cell Penetrating Peptides: Limits versus Prospects. J. Pharm. Sci..

[82] Tacken P., Joosten B., Reddy A., Wu D., Eek A., Laverman P., Kretz-Rommel A., Adema G., Torensma R., Figdor C. (2008). No Advantage of Cell-Penetrating Peptides over Receptor-Specific Antibodies in Targeting Antigen to Human Dendritic Cells for Cross-Presentation. J. Immunol..

[83] Perham R. (2000). Swinging Arms and Swinging Domains in Multifunctional Enzymes: Catalytic Machines for Multistep Reactions. Annu. Rev. Biochem..

[84] Trovato M. (2012). Delivery strategies for novel vaccine formulations. World J. Virol..

[85] Domingo G., Orru S., Perham R. (2001). Multiple Display of Peptides and Proteins on a Macromolecular Scaffold Derived from a Multienzyme Complex. J. Mol. Biol..

[86] Domingo G., Caivano A., Sartorius R., Barba P., Bäckström M., Piatier-Tonneau D., Guardiola J., De Berardinis P., Perham R. (2003). Induction of specific T-helper and cytolytic responses to epitopes displayed on a virus-like protein scaffold derived from the pyruvate dehydrogenase multienzyme complex. Vaccine.

[87] D’Apice L., Sartorius R., Caivano A., Mascolo D., Del Pozzo G., Di Mase D., Ricca E., Pira G., Manca F., Malanga D. (2007). Comparative analysis of new innovative vaccine formulations based on the use of procaryotic display systems. Vaccine.

